# Poly[[diaqua-μ_4_-tartrato-μ_2_-tartrato-dimanganese(II)] dihydrate]

**DOI:** 10.1107/S1600536807067839

**Published:** 2008-01-16

**Authors:** Chunhua Ge, Zhen Zhao, Guangxi Han, Xiangdong Zhang

**Affiliations:** a College of Chemistry, Liaoning University, Shenyang 110036, People’s Republic of China; b Department of Chemistry, Anshan Normal University, Anshan 114007, People’s Republic of China

## Abstract

In the title compound, {[Mn(C_4_H_4_O_6_)(H_2_O)]·H_2_O}_*n*_, the Mn^2+^ ion is connected to three different tartrate anions and a water mol­ecule, resulting in a distorted MnO_6_ octa­hedral geometry. There are two tartrate half-anions in the asymmetric unit, both of which are completed by crystallographic twofold rotation symmetry. The tartrate dianions bridge the Mn^2+^ ions to form a wave-like infinite layer. A series of O—H⋯O hydrogen bonds link the layers into a three-dimensional network.

## Related literature

For related literature, see: Kam *et al.* (2007 ▶).
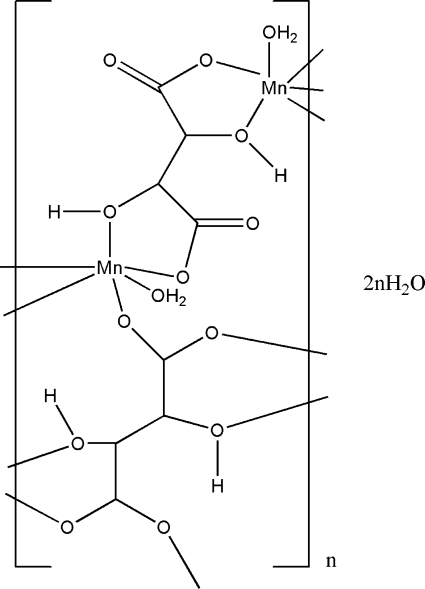


## Experimental

### 

#### Crystal data

[Mn(C_4_H_4_O_6_)(H_2_O)]·H_2_O
*M* *_r_* = 239.04
Monoclinic, 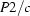

*a* = 11.029 (3) Å
*b* = 7.3925 (18) Å
*c* = 10.165 (3) Å
β = 112.149 (3)°
*V* = 767.6 (3) Å^3^
*Z* = 4
Mo *K*α radiation
μ = 1.74 mm^−1^
*T* = 293 (2) K
0.25 × 0.20 × 0.18 mm

#### Data collection

Bruker SMART CCD diffractometer
Absorption correction: multi-scan (*SADABS*; Bruker, 2001 ▶) *T*_min_ = 0.661, *T*_max_ = 0.739
3884 measured reflections
1507 independent reflections
1481 reflections with *I* > 2σ(*I*)
*R*_int_ = 0.012

#### Refinement

*R*[*F* ^2^ > 2σ(*F* ^2^)] = 0.027
*wR*(*F* ^2^) = 0.074
*S* = 1.10
1507 reflections
118 parameters
H-atom parameters constrained
Δρ_max_ = 0.45 e Å^−3^
Δρ_min_ = −0.69 e Å^−3^

### 

Data collection: *SMART* (Bruker, 2001 ▶); cell refinement: *SAINT* (Bruker, 2001 ▶); data reduction: *SAINT*; program(s) used to solve structure: *SHELXS97* (Sheldrick, 1997*a* ▶); program(s) used to refine structure: *SHELXL97* (Sheldrick, 1997*a* ▶); molecular graphics: *SHELXTL* (Sheldrick, 1997*b* ▶); software used to prepare material for publication: *SHELXL97*.

## Supplementary Material

Crystal structure: contains datablocks I, global. DOI: 10.1107/S1600536807067839/hb2676sup1.cif

Structure factors: contains datablocks I. DOI: 10.1107/S1600536807067839/hb2676Isup2.hkl

Additional supplementary materials:  crystallographic information; 3D view; checkCIF report

## Figures and Tables

**Table 1:** Selected bond lengths (Å)

Mn1—O6	2.1036 (15)
Mn1—O1	2.1444 (15)
Mn1—O5^i^	2.1695 (15)
Mn1—O1*W*	2.2018 (16)
Mn1—O3	2.2230 (14)
Mn1—O4^i^	2.2518 (14)

Symmetry code: (i) 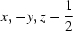.

**Table 2:** Hydrogen-bond geometry (Å, °)

*D*—H⋯*A*	*D*—H	H⋯*A*	*D*⋯*A*	*D*—H⋯*A*
O4—H4⋯O2*W*^ii^	0.82	1.81	2.628 (2)	175
O3—H3*A*⋯O2^iii^	0.82	1.75	2.561 (2)	173
O1*W*—H1*WA*⋯O2^iv^	0.82	2.04	2.643 (2)	130
O2*W*—H2*WA*⋯O5^v^	0.82	2.29	2.895 (2)	131
O2*W*—H2*WB*⋯O4^i^	0.82	2.25	2.919 (3)	140

Symmetry codes: (i) 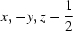; (ii) 
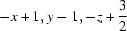
; (iii) 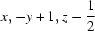; (iv) 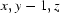; (v) 
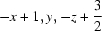
.

**Fig. 1.**
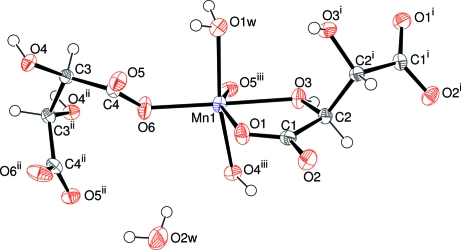
View of (I), showing displacement ellipsoids drawn at 50% probability level (arbitrary spheres for the H atoms). Symmetry codes: (i) -x, y, 1/2 - z; (ii) 1 - x, y, 3/2 - z; (iii) x, -y, z - 1/2.

**Fig. 2.**
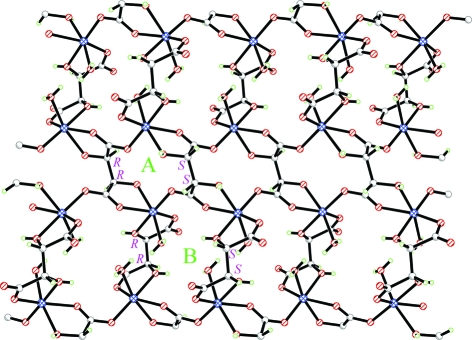
View of the layered network in (I) along [010] direction, with the A and B rings indicated (see text).

## supplementary crystallographic information

### Comment

Researchers have been interested in the study of tartrate-based coordination polymers, which has resulted in the formation of many interesting structures (*e.g.* Kam *et al.*, 2007). The title compound, (I), is centrosymmetric (Fig. 1). The Mn(II) ion adopts a distorted MnO_6_ octahedral geometry (Table 1).

In the crystal, one (*R,R*) and one (*S,S*) tartrate ligands coordinate with two metal ions to form a 'tetrameric' A ring (Fig. 2). Then, two (*R,R*), two (*S,S*) tartrate ligands and four metal ions form 'hexameric' B ring (Fig. 2). Overal, a layered, two-dimensional, coordination polymer arises. The layers encompass small channels occupied by the uncoordinated water molecules, which interact with the layers by way of O—H···O hydrogen bonds (Table 2).

### Experimental

A mixture of aqueous Mn(NO_3_)_2_ (2 mmol), racemic tartaric acid (2 mmol) and NaOH (4 mmol) in 20 ml water was stirred for 2 h. The resulting solution was filtered and allowed to stand in air. Slow evaporation at room temperature for several weeks yielded yellow blocks of (I).

### Refinement

The H atoms were located in a different map, relocated in idealized positions (C—H = 0.98 Å, O—H = 0.82 Å) and refined as riding with *U*_iso_(H) = 1.2*U*_eq_(C) or 1.5*U*_eq_(O).

### Crystal data

**Table:**

[Mn(C_4_H_4_O_6_)(H_2_O)]·H_2_O	*F*_000_ = 484
*M**_r_* = 239.04	*D*_x_ = 2.068 Mg m^−^^3^
Monoclinic, *P*2/*c*	Mo *K*α radiation λ = 0.71073 Å
Hall symbol: -P 2yc	Cell parameters from 456 reflections
*a* = 11.029 (3) Å	θ = 2.8–22.3º
*b* = 7.3925 (18) Å	µ = 1.74 mm^−^^1^
*c* = 10.165 (3) Å	*T* = 293 (2) K
β = 112.149 (3)º	Block, yellow
*V* = 767.6 (3) Å^3^	0.25 × 0.20 × 0.18 mm
*Z* = 4	

### Data collection

**Table:**

Bruker SMART CCD diffractometer	1507 independent reflections
Radiation source: fine-focus sealed tube	1481 reflections with *I* > 2σ(*I*)
Monochromator: graphite	*R*_int_ = 0.012
*T* = 293(2) K	θ_max_ = 26.0º
ω scans	θ_min_ = 2.0º
Absorption correction: multi-scan(SADABS; Bruker, 2001)	*h* = −12→13
*T*_min_ = 0.661, *T*_max_ = 0.739	*k* = −9→5
3884 measured reflections	*l* = −12→12

### Refinement

**Table:**

Refinement on *F*^2^	Secondary atom site location: difference Fourier map
Least-squares matrix: full	Hydrogen site location: inferred from neighbouring sites
*R*[*F*^2^ > 2σ(*F*^2^)] = 0.027	H-atom parameters constrained
*wR*(*F*^2^) = 0.074	*w* = 1/[σ^2^(*F*_o_^2^) + (0.0399*P*)^2^ + 0.6888*P*] where *P* = (*F*_o_^2^ + 2*F*_c_^2^)/3
*S* = 1.11	(Δ/σ)_max_ < 0.001
1507 reflections	Δρ_max_ = 0.45 e Å^−^^3^
118 parameters	Δρ_min_ = −0.69 e Å^−^^3^
Primary atom site location: structure-invariant direct methods	Extinction correction: none

### Special details

**Table:**

Geometry. All e.s.d.'s (except the e.s.d. in the dihedral angle between two l.s. planes) are estimated using the full covariance matrix. The cell e.s.d.'s are taken into account individually in the estimation of e.s.d.'s in distances, angles and torsion angles; correlations between e.s.d.'s in cell parameters are only used when they are defined by crystal symmetry. An approximate (isotropic) treatment of cell e.s.d.'s is used for estimating e.s.d.'s involving l.s. planes.
Refinement. Refinement of *F*^2^ against ALL reflections. The weighted *R*-factor *wR* and goodness of fit *S* are based on *F*^2^, conventional *R*-factors *R* are based on *F*, with *F* set to zero for negative *F*^2^. The threshold expression of *F*^2^ > σ(*F*^2^) is used only for calculating *R*-factors(gt) *etc*. and is not relevant to the choice of reflections for refinement. *R*-factors based on *F*^2^ are statistically about twice as large as those based on *F*, and *R*- factors based on ALL data will be even larger.

### Fractional atomic coordinates and isotropic or equivalent isotropic displacement parameters (Å^2^)

**Table:**

	*x*	*y*	*z*	*U*_iso_*/*U*_eq_	
Mn1	0.25422 (3)	0.14754 (4)	0.41123 (3)	0.01934 (13)	
C1	0.13897 (18)	0.5035 (3)	0.43625 (19)	0.0179 (4)	
C2	0.07525 (17)	0.4815 (2)	0.27492 (18)	0.0162 (4)	
H2	0.1024	0.5821	0.2294	0.019*	
C3	0.42697 (17)	−0.2562 (3)	0.74009 (19)	0.0181 (4)	
H3	0.3829	−0.3503	0.6706	0.022*	
C4	0.35823 (17)	−0.0765 (3)	0.68319 (19)	0.0200 (4)	
O1	0.20895 (14)	0.3801 (2)	0.51027 (14)	0.0251 (3)	
O2	0.11465 (16)	0.65022 (18)	0.48441 (15)	0.0242 (3)	
O3	0.11895 (14)	0.31700 (19)	0.23691 (14)	0.0212 (3)	
H3A	0.1245	0.3256	0.1590	0.032*	
O4	0.41270 (13)	−0.30125 (19)	0.87015 (14)	0.0206 (3)	
H4	0.4041	−0.4114	0.8703	0.031*	
O5	0.28552 (14)	−0.00821 (19)	0.73884 (15)	0.0241 (3)	
O6	0.37862 (15)	−0.0127 (2)	0.57879 (16)	0.0315 (4)	
O1W	0.08037 (15)	−0.0100 (2)	0.39729 (17)	0.0306 (3)	
H1WA	0.0662	−0.1156	0.3707	0.046*	
H1WB	0.0915	−0.0298	0.4808	0.046*	
O2W	0.6263 (2)	0.3474 (2)	0.6453 (2)	0.0511 (5)	
H2WA	0.6577	0.2909	0.7200	0.077*	
H2WB	0.5735	0.2819	0.5859	0.077*	

### Atomic displacement parameters (Å^2^)

**Table:**

	*U*^11^	*U*^22^	*U*^33^	*U*^12^	*U*^13^	*U*^23^
Mn1	0.02154 (19)	0.01935 (19)	0.01749 (19)	0.00330 (10)	0.00775 (13)	0.00311 (10)
C1	0.0182 (8)	0.0198 (9)	0.0160 (9)	−0.0034 (7)	0.0067 (7)	−0.0012 (7)
C2	0.0180 (9)	0.0170 (9)	0.0139 (8)	0.0015 (7)	0.0064 (7)	0.0005 (7)
C3	0.0175 (9)	0.0200 (9)	0.0165 (8)	−0.0014 (7)	0.0060 (7)	0.0010 (7)
C4	0.0158 (8)	0.0228 (10)	0.0182 (9)	−0.0014 (7)	0.0028 (7)	0.0040 (7)
O1	0.0303 (8)	0.0254 (7)	0.0158 (7)	0.0064 (6)	0.0045 (6)	0.0005 (5)
O2	0.0358 (8)	0.0200 (7)	0.0177 (7)	0.0007 (6)	0.0110 (6)	−0.0020 (5)
O3	0.0274 (7)	0.0235 (7)	0.0143 (6)	0.0079 (6)	0.0095 (5)	0.0012 (5)
O4	0.0242 (7)	0.0193 (7)	0.0208 (7)	0.0014 (5)	0.0112 (5)	0.0051 (5)
O5	0.0274 (7)	0.0215 (7)	0.0261 (7)	0.0038 (6)	0.0131 (6)	0.0038 (5)
O6	0.0261 (7)	0.0416 (9)	0.0294 (8)	0.0111 (6)	0.0136 (6)	0.0194 (7)
O1W	0.0302 (8)	0.0250 (8)	0.0336 (8)	−0.0013 (6)	0.0086 (6)	0.0057 (6)
O2W	0.0643 (13)	0.0254 (9)	0.0473 (12)	0.0007 (8)	0.0024 (10)	0.0043 (7)

### Geometric parameters (Å, °)

**Table:**

Mn1—O6	2.1036 (15)	C3—C4	1.530 (3)
Mn1—O1	2.1444 (15)	C3—C3^iii^	1.546 (3)
Mn1—O5^i^	2.1695 (15)	C3—H3	0.9800
Mn1—O1W	2.2018 (16)	C4—O5	1.249 (2)
Mn1—O3	2.2230 (14)	C4—O6	1.257 (2)
Mn1—O4^i^	2.2518 (14)	O3—H3A	0.8199
C1—O1	1.247 (2)	O4—Mn1^iv^	2.2518 (14)
C1—O2	1.260 (2)	O4—H4	0.8198
C1—C2	1.530 (2)	O5—Mn1^iv^	2.1695 (15)
C2—O3	1.415 (2)	O1W—H1WA	0.8215
C2—C2^ii^	1.542 (3)	O1W—H1WB	0.8237
C2—H2	0.9800	O2W—H2WA	0.8201
C3—O4	1.429 (2)	O2W—H2WB	0.8201
			
O6—Mn1—O1	105.52 (6)	C2^ii^—C2—H2	109.1
O6—Mn1—O5^i^	97.63 (6)	O4—C3—C4	109.91 (15)
O1—Mn1—O5^i^	153.64 (6)	O4—C3—C3^iii^	110.62 (18)
O6—Mn1—O1W	92.32 (6)	C4—C3—C3^iii^	113.12 (11)
O1—Mn1—O1W	95.92 (6)	O4—C3—H3	107.7
O5^i^—Mn1—O1W	95.55 (6)	C4—C3—H3	107.7
O6—Mn1—O3	178.49 (5)	C3^iii^—C3—H3	107.7
O1—Mn1—O3	73.58 (5)	O5—C4—O6	125.50 (19)
O5^i^—Mn1—O3	83.51 (5)	O5—C4—C3	119.39 (16)
O1W—Mn1—O3	86.58 (6)	O6—C4—C3	115.08 (17)
O6—Mn1—O4^i^	96.86 (6)	C1—O1—Mn1	120.25 (12)
O1—Mn1—O4^i^	90.96 (6)	C2—O3—Mn1	117.52 (10)
O5^i^—Mn1—O4^i^	73.67 (5)	C2—O3—H3A	110.8
O1W—Mn1—O4^i^	166.64 (6)	Mn1—O3—H3A	122.8
O3—Mn1—O4^i^	84.40 (5)	C3—O4—Mn1^iv^	114.70 (10)
O1—C1—O2	124.68 (17)	C3—O4—H4	106.7
O1—C1—C2	119.90 (16)	Mn1^iv^—O4—H4	113.7
O2—C1—C2	115.41 (16)	C4—O5—Mn1^iv^	119.92 (12)
O3—C2—C1	108.55 (14)	C4—O6—Mn1	128.71 (13)
O3—C2—C2^ii^	110.22 (11)	Mn1—O1W—H1WA	125.2
C1—C2—C2^ii^	110.78 (18)	Mn1—O1W—H1WB	103.9
O3—C2—H2	109.1	H1WA—O1W—H1WB	96.1
C1—C2—H2	109.1	H2WA—O2W—H2WB	108.4

Symmetry codes: (i) *x*, −*y*, *z*−1/2; (ii) −*x*, *y*, −*z*+1/2; (iii) −*x*+1, *y*, −*z*+3/2; (iv) *x*, −*y*, *z*+1/2.

### Hydrogen-bond geometry (Å, °)

**Table:**

*D*—H···*A*	*D*—H	H···*A*	*D*···*A*	*D*—H···*A*
O4—H4···O2W^v^	0.82	1.81	2.628 (2)	175
O3—H3A···O2^vi^	0.82	1.75	2.561 (2)	173
O1W—H1WA···O2^vii^	0.82	2.04	2.643 (2)	130
O2W—H2WA···O5^iii^	0.82	2.29	2.895 (2)	131
O2W—H2WB···O4^i^	0.82	2.25	2.919 (3)	140

Symmetry codes: (v) −*x*+1, *y*−1, −*z*+3/2; (vi) *x*, −*y*+1, *z*−1/2; (vii) *x*, *y*−1, *z*; (iii) −*x*+1, *y*, −*z*+3/2; (i) *x*, −*y*, *z*−1/2.
